# Predictive and prognostic value of preoperative pan-immune-inflammation value in patients with locally advanced rectal cancer

**DOI:** 10.17305/bb.2024.10658

**Published:** 2024-08-30

**Authors:** Peipei Shen, Yu Xu, Jiahao Zhu, Danqi Qian, Bo Yang, Yong Mao, Shengjun Ji, Ke Gu, Yutian Zhao

**Affiliations:** 1 Department of Radiotherapy and Oncology, The Affiliated Hospital of Jiangnan University, Wuxi, China; 2 Wuxi Clinical Cancer Center, Wuxi, China; 3 Department of Oncology, The Affiliated Hospital of Jiangnan University, Wuxi, China; 4 Department of Radiotherapy and Oncology, The Affiliated Suzhou Hospital of Nanjing Medical University, Gusu School, Nanjing Medical University, Suzhou, China

**Keywords:** Pan-immune-inflammation value (PIV), locally advanced rectal cancer (LARC), inflammation indicators, neoadjuvant chemoradiotherapy (nCRT), prognosis

## Abstract

This study aimed to investigate the prognostic value of the pan-immune-inflammation value (PIV) in patients with locally advanced rectal cancer (LARC) who received neoadjuvant chemoradiotherapy (nCRT) followed by total mesorectal excision. We retrospectively collected and analyzed the clinicopathological data of 215 resected LARC patients. X-tile software was used to determine the optimal threshold value for PIV in predicting overall survival (OS). The predictive ability of PIV for pathological complete regression (pCR), OS, and disease-free survival (DFS) was evaluated and compared with other inflammation markers. Univariate and multivariate logistic regression analyses for pCR and Cox regression analyses for OS and DFS were conducted. The optimal threshold value for PIV was determined to be 454.7 based on the X-tile software. Patients were then categorized into low (≤ 454.7) and high (> 454.7) PIV groups comprising 153 and 62 patients, respectively. PIV demonstrated superior predictive ability for pCR, OS, and DFS compared to other inflammation markers. LARC patients with low PIV had significantly higher pCR (*P* ═ 0.029), OS (*P* ═ 0.002), and DFS (*P* ═ 0.001) rates compared to those with high PIV. Multivariate regression analysis identified PIV as an independent prognostic factor for pCR (odds ratio ═ 0.32; 95% confidence interval [CI], 0.10–0.80; *P* ═ 0.014), OS (hazard ratio ═ 3.08; 95% CI, 1.77–5.35; *P* ═ 0.001), and DFS (hazard ratio ═ 2.53; 95% CI, 1.58–4.06; *P* ═ 0.002). This study confirmed that preoperative PIV could serve as a useful independent prognostic factor in LARC patients treated with nCRT.

## Introduction

Colorectal cancer (CRC) ranks as the third most prevalent malignant neoplasm globally and stands as the third leading cause of tumor-related mortality, posing a significant threat to human health and survival [1]. Rectal cancer accounts for approximately 30% of all CRC cases, with around 65% of newly diagnosed rectal cancer patients already in advanced stages [2]. The standard treatment for locally advanced rectal cancer (LARC) involves preoperative chemoradiotherapy, followed by total mesorectal excision. Although the conventional neoadjuvant treatment regimen introduced in 2004, as well as more recent total neoadjuvant therapy (TNT) modalities, have substantially reduced local recurrence in LARC, approximately 25% of cases still develop distant metastasis after surgery [3, 4]. Moreover, the improved rate of pathological complete response (pCR) has not translated into significant survival benefits for patients. Therefore, more accurate prognostic indicators based on preoperative clinical parameters are needed to stratify high-risk LARC patients and guide individualized treatment approaches. The link between systemic inflammation and cancer prognosis has been extensively studied [5–7]. Increased neutrophil production in the presence of inflammation has been shown to promote tumor initiation, growth, and metastasis. Elevated platelet counts and activity have also been observed in cancer patients, contributing to tumor cell proliferation and extravasation. The role of lymphocytes in tumor progression is more complex, with different subsets exerting contrasting effects. In a study by Zhang et al., the prognostic value of inflammatory markers was specifically investigated in a large cohort of CRC patients [8]. The study found that the neutrophil-to-lymphocyte ratio (NLR) was an effective biomarker and an independent predictor for both disease-free survival (DFS) and overall survival (OS). NLR, calculated by dividing the neutrophil count by the lymphocyte count, reflects the balance between proinflammatory neutrophils and anti-tumor lymphocytes. An elevated NLR suggests a heightened systemic inflammatory state and has been associated with poorer outcomes in various cancers, including CRC. These findings underscore the importance of considering systemic inflammatory markers, such as NLR, in assessing the prognosis of cancer patients and identifying those at higher risk of recurrence and mortality. Given the complex relationship between immunity, inflammation, and cancer, composite inflammatory biomarkers that reflect the systemic immune-inflammatory state may have greater prognostic potential. Recently, a more comprehensive index called the pan-immune-inflammation value (PIV) has been proposed, incorporating four parameters: neutrophil, platelet, monocyte, and lymphocyte counts [9]. PIV has demonstrated superior prognostic and predictive capabilities in several cancers, including esophageal squamous cell carcinoma, breast cancer, prostate cancer, and lung cancer [10–14]. However, the prognostic value of PIV in LARC patients receiving neoadjuvant chemoradiotherapy (nCRT) remains understudied. The aim of this study is to evaluate the prognostic significance of PIV in LARC patients undergoing nCRT. We seek to determine whether PIV can serve as a predictive factor for tumor response to neoadjuvant treatment, as well as survival outcomes and metastasis. Furthermore, we will compare PIV with other inflammatory markers to assess its predictive efficacy.

**Figure 1.**
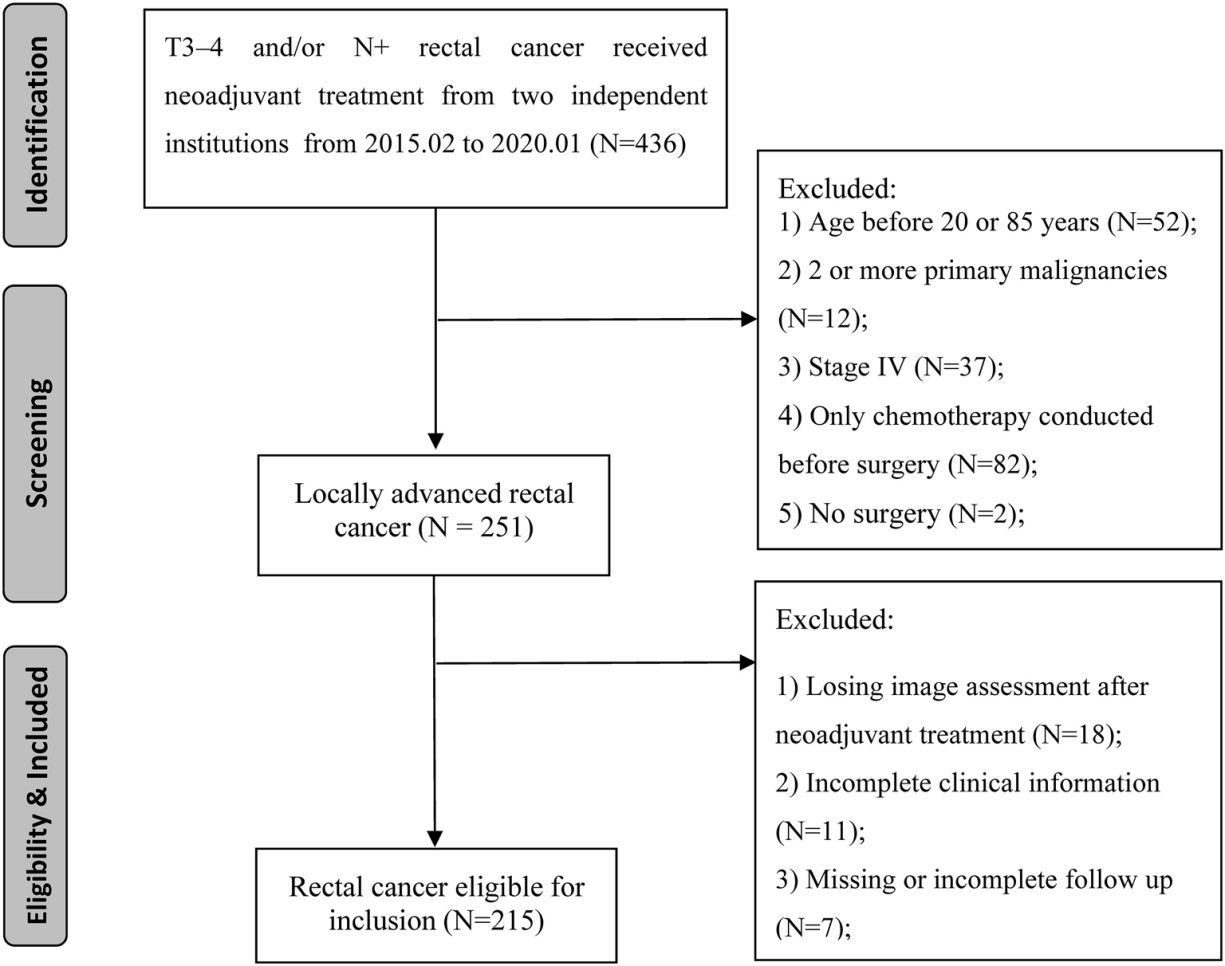
**Flowchart of patient inclusion and exclusion**.

## Materials and methods

### Patients

A total of 215 LARC patients who received nCRT followed by surgery at The Affiliated Hospital of Jiangnan University and The Affiliated Suzhou Hospital of Nanjing Medical University between February 2015 and January 2020 were retrospectively analyzed. The following selection criteria were applied: (1) Clinical stage II–III (cT3-4 and/or cN1-2) classified by magnetic resonance imaging (MRI); (2) age 20–85 years; (3) adenocarcinoma or mucinous adenocarcinoma; (4) rectal cancer as the primary and only diagnosed cancer; and (5) all patients received nCRT followed by radical surgery. The exclusion criteria were as follows: (1) patients who received corticosteroids, albumin, statins, or nutritional therapy during treatment; (2) missing data for analysis; and (3) surgery performed at another hospital. Figure 1 presents the patient selection flowchart. The review of data for this study was approved by the institutional review boards of The Affiliated Hospital of Jiangnan University and The Affiliated Suzhou Hospital of Nanjing Medical University. This study adheres to the principles of the Declaration of Helsinki.

**Table 1:** Characteristics of 215 LARC patients grouped by PIV

**Characteristics**	**Total (*n* ═ 215)**	**Low-PIV (*n* ═ 153)**	**High-PIV (*n* ═ 62)**	***P* value**
Age (years)				0.243
< 60	114 (53.0%)	85 (55.6%)	29 (46.8%)	
≥ 60	101 (47.0%)	68 (44.4%)	33 (53.2%)	
Gender				0.742
Male	132 (61.4%)	95 (62.1%)	37 (59.7%)	
Female	83 (38.6%)	58 (37.9%)	25 (40.3%)	
Differentiation				0.891
Well	21 (9.7%)	14 (9.2%)	7 (11.3%)	
Moderate	173 (80.5%)	124 (81.0%)	49 (79.0%)	
Poor	21 (9.8%)	15 (9.8%)	6 (9.7%)	
CEA pretreatment				0.627
Normal	120 (55.8%)	87 (56.9%)	33 (53.2%)	
Elevated	95 (44.2%)	66 (43.1%)	29 (46.8%)	
cT				0.859
cT3	152 (70.7%)	117 (76.5%)	46 (74.2%)	
cT4	63 (29.3%)	36 (23.5%)	16 (25.8%)	
cN				0.752
Negative	66 (30.7%)	46 (30.1%)	20 (32.3%)	
Positive	149 (69.3%)	107 (69.9%)	42 (67.7%)	
cTNM				0.752
II	66 (30.7%)	46 (30.1%)	20 (32.3%)	
III	149 (69.3%)	107 (69.9%)	42 (67.7%)	
ypT				0.101
ypT0	38 (17.7%)	33 (21.6%)	5 (8.1%)	
ypT1-2	47 (21.8%)	34 (22.2%)	13 (21.0%)	
ypT3-4	130 (60.5%)	86 (56.2%)	44 (70.9%)	
ypN				0.012
Negative	155 (72.1%)	119 (77.8%)	36 (58.1%)	
Positive	60 (27.9%)	34 (22.2%)	26 (41.9%)	
NLR				<0.001
Mean ± SD	2.59 ± 1.45	1.98 ± 0.73	4.11 ± 1.66	
MLR				<0.001
Mean ± SD	0.31 ± 0.17	0.24 ± 0.09	0.48 ± 0.21	
PLR				<0.001
Mean ± SD	158 ± 79	128 ± 49	233 ± 89	
SII				<0.001
Mean ± SD	713 ± 515	476 ± 195	1299 ± 588	

CEA: Carcinoembryonic antigen; NLR: Neutrophil-to-lymphocyte ratio; MLR: Monocyte-to-lymphocyte ratio; PIV: Pan-immune-inflammation value; PLR: Platelet-to-lymphocyte ratio; SD: Standard deviation; SII: Systemic index of inflammation; LARC: Locally advanced rectal cancer.

### Pathology assessment and survival outcome definition

In accordance with recommendations from the National Comprehensive Cancer Network (NCCN), the American Joint Committee on Cancer (AJCC) 8th edition staging system was used to determine the pathological stage of cancer patients. pCR is defined as the absence of detectable residual tumor cells in both the primary tumor site and the resected lymph nodes within the surgical specimens. The survival outcomes assessed in this study include OS and DFS. OS is defined as the time from surgery to death or the last follow-up visit, while DFS represents the time from surgery to the first recurrence or cancer-related death. The last follow-up was conducted in May 2023.

**Figure 2.**
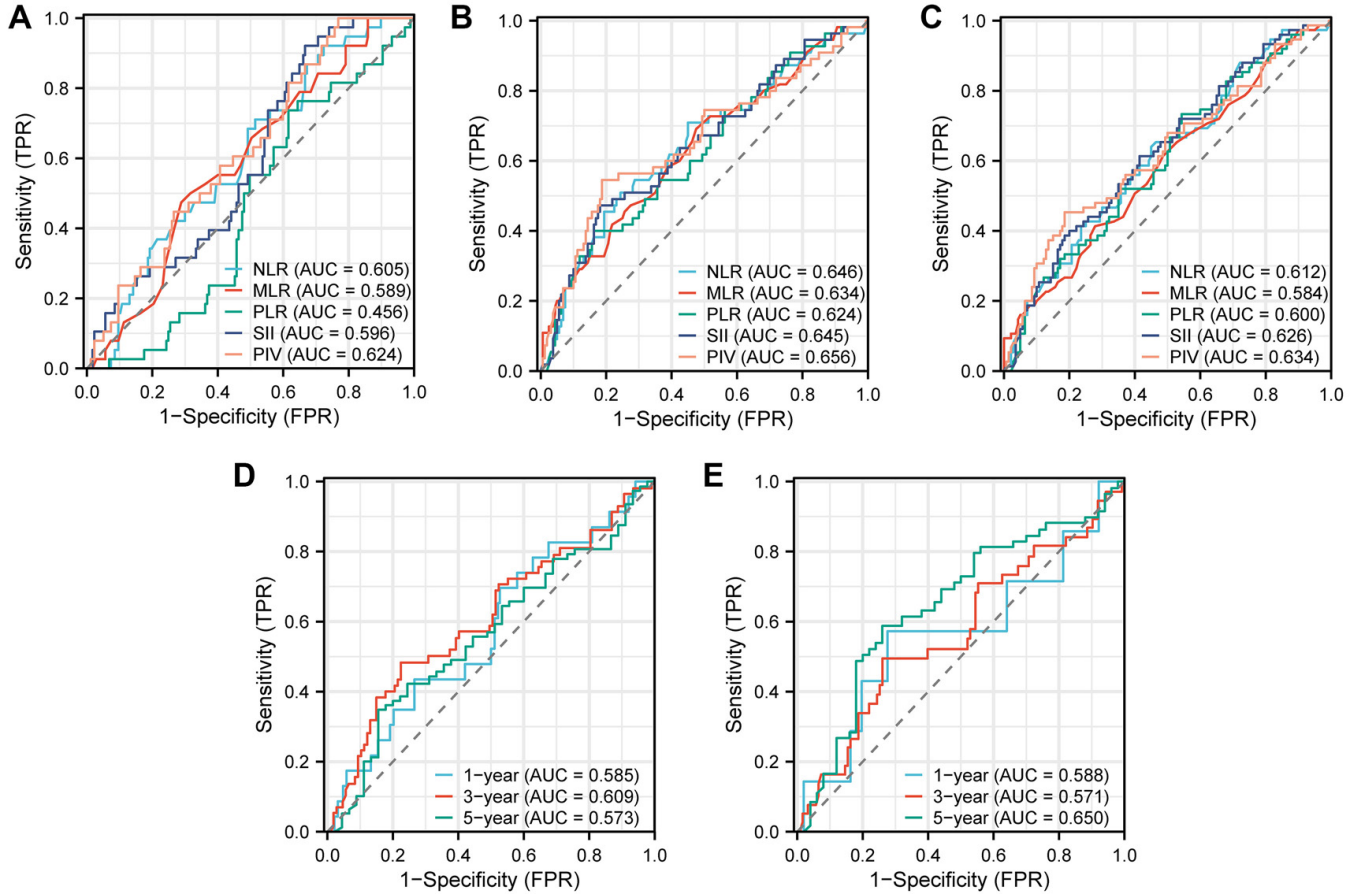
**AUC comparisons between PIV and other inflammation indicators by ROC.** (A) pCR; (B) OS; (C) DFS. The prognostic value of PIV in time-dependent ROC; (D) OS; (E) DFS. ROC: Receiver operating characteristic; NLR: Neutrophil-to-lymphocyte ratio; MLR: Monocyte-to-lymphocyte ratio; PIV: Pan-immune-inflammation value; PLR: Platelet-to-lymphocyte ratio; SII: Systemic index of inflammation; AUC: Areas under the curve; OS: Overall survival; pCR: Pathological complete response; DFS: Disease-free survival.

### Definition of PIV and other inflammation markers

Blood counts obtained within two weeks before the start of nCRT were extracted from the hospitals’ clinical data repositories. The absolute counts of neutrophils, monocytes, platelets, and lymphocytes were used to derive additional parameters, such as the platelet-to-lymphocyte ratio (PLR), NLR, monocyte-to-lymphocyte ratio (MLR), and systemic index of inflammation (SII). Additionally, the platelet, neutrophil, and monocyte counts were multiplied together and divided by the lymphocyte count to calculate the PIV. X-tile software was used to define the optimal PIV cutoff according to OS [15]. Detailed information on the calculation formulas for PLR, NLR, MLR, and SII can be found in Table S1.

### Propensity score matching

Since this study was a retrospective analysis, there was a potential for imbalanced characteristics between the Low- and High-PIV groups. To address this, we conducted propensity score matching using nearest-neighbor matching with a caliper of 0.05. Patients from the Low- and High-PIV groups were matched in a 1:1 ratio. Propensity score matching and multivariable analysis are valuable methods for making such comparisons.

### Ethical statement

The study was conducted at The Affiliated Hospital of Jiangnan University and The Affiliated Suzhou Hospital of Nanjing Medical University in accordance with the Declaration of Helsinki. All participants provided informed consent.

### Statistical analysis

For categorical variables, chi-squared tests or Fisher’s exact test were used. For continuous variables, a *t*-test was performed. To compare the performance of PIV with other inflammation-based markers, receiver operating characteristic (ROC) curves were employed, and the areas under the curves (AUCs) were compared to evaluate the discriminatory ability of PIV compared to other markers. OS and DFS rates were calculated using the Kaplan–Meier method, with differences assessed using log-rank tests. Univariate and multivariate logistic regression analyses were performed to explore associations between pCR and prognostic factors. A Cox proportional hazards regression model was used to analyze the relationship between OS, DFS, and prognostic factors in both univariate and multivariate analyses. Factors with *P* < 0.1 in univariate analyses were included in multivariate models. Statistical significance was defined as a two-sided *P* < 0.05. All statistical analyses were performed using R software version 4.2.1.

## Results

### Patient characteristics

Table 1 presents the fundamental characteristics of the patients included in this investigation. The study comprised a total of 215 patients diagnosed with LARC, consisting of 132 male and 83 female patients. The average age at diagnosis was 58 years, ranging from 25 to 79 years. The mean value of NLR was 2.59 (ranging from 0.38 to 9.52). The mean value of MLR was 0.31 (ranging from 0.06 to 1.25). The mean value of PLR was 158 (ranging from 38 to 502). The mean value of SII was 713 (ranging from 94 to 2751). The mean value of PIV was 397 (ranging from 38 to 1818).

**Figure 3.**
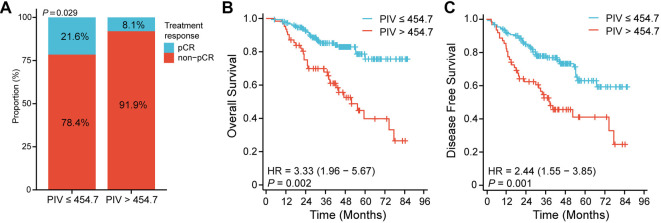
**Differences in pCR (A), OS (B), and DFS (C) between low-PIV and high-PIV LARC patients treated with nCRT.** PIV: Pan-immune-inflammation value; nCRT: Neoadjuvant chemoradiotherapy; LARC: Locally advanced rectal cancer; OS: Overall survival; pCR: Pathological complete response; DFS: Disease-free survival.

**Table 2:** Univariate and multivariate logistic analysis for pCR in LARC

**Characteristic**	**Univariable (pCR)**		**Multivariable (pCR)**	
	**HR (95% CI)**	***P***	**HR (95% CI)**	***P***
*Age*				
< 60	ref			
≥ 60	0.90 (0.44–1.81)	0.76		
*Gender*				
Male	ref			
Female	1.55 (0.76–3.15)	0.223		
*Differentiation*				
Well	ref			
Moderate	0.73 (0.26–2.35)	0.559		
Poor	0.16 (0.01–1.12)	0.111		
*CEA pretreatment*				
Normal	ref			
Elevated	0.79 (0.38–1.60)	0.521		
*cT*				
cT3	ref			
cT4	0.70 (0.20–3.23)	0.601		
*cN*				
Negative	ref		ref	
Positive	2.21 (0.97–5.74)	0.076	2.21 (0.96–5.77)	0.079
*PIV*				
≤ 454.7	ref		ref	
> 454.7	0.32 (0.10–0.79)	0.024	0.32 (0.10–0.80)	0.014

CEA: Carcinoembryonic antigen; pCR: Pathologic complete response; PIV: Pan-immune-inflammation value; ref: Reference; LARC: Locally advanced rectal cancer.

### Prognostic comparison between PIV and other inflammation indicators

ROC analyses comparing the prognostic value of PIV with other inflammation indicators, such as NLR, MLR, PLR, and SII were performed. PIV was found to have the largest AUC and demonstrated superior prognostic ability compared to other inflammation indicators in predicting pCR, OS, and DFS (Figure 2A–2C). The time-dependent ROC curve is shown in Figure 2D and 2E.

### Optimal threshold value of PIV identification

Based on the X-tile binary classification, 454.7 was determined as the optimal cutoff value of PIV, and patients were divided into the low (≤ 454.7) and high (> 454.7) PIV groups. Differences in clinicopathological characteristics between the two groups are shown in Table 1. Significant differences were observed in ypN, NLR, MLR, PLR, and SII. Figure 3A illustrates the difference in pCR ratio between the two groups. Additionally, LARC patients in the low-PIV group were found to have better OS (*P* ═ 0.002) and DFS (*P* ═ 0.001). The Kaplan–Meier curves for OS and DFS are presented in Figure 3B and 3C.

### Logistic and Cox outcomes for independent prognostic factors before and after propensity score matching

Before propensity score matching, univariate and multivariate logistic analyses for pCR showed that clinical lymph node metastasis state (cN) and PIV were important predictors for pCR, with PIV serving as an independent predictor (odds ratio ═ 0.32; 95% CI, 0.10–0.80; *P* ═ 0.014) (Table 2). Univariate Cox analyses for OS (Table 3) and DFS (Table 4) revealed that cN, ypN, and PIV were significant predictors for OS, while cN, ypT, ypN, and PIV were important predictors for DFS. Multivariate Cox analyses demonstrated that PIV (hazard ratio [HR] ═ 3.08; 95% confidence interval [CI], 1.77–5.35; *P* ═ 0.001) and ypN (HR ═ 2.11; 95% CI, 1.21–3.57; *P* ═ 0.019) were independent predictors for OS, and PIV (HR ═ 2.53; 95% CI, 1.58–4.06; *P* ═ 0.002) and cN (HR ═ 2.21; 95% CI, 1.26–3.87; *P* ═ 0.006) were independent predictors for DFS.

**Table 3:** Univariate and multivariate Cox analysis for OS in LARC

**Characteristic**	**Univariable (OS)**		**Multivariable (OS)**	
	**HR (95% CI)**	***P***	**HR (95% CI)**	***P***
*Age*				
< 60	ref			
≥ 60	1.28 (0.75--2.17)	0.368		
*Gender*				
Male	ref			
Female	0.96 (0.55--1.66)	0.873		
*Differentiation*				
Well	ref			
Moderate	1.03 (0.41--2.60)	0.954		
Poor	2.02 (0.66--6.19)	0.219		
*CEA pretreatment*				
Normal	ref			
Elevated	1.18 (0.70--2.01)	0.539		
*cT*				
cT1-2	ref			
cT3-4	0.95 (0.30--3.06)	0.936		
*cN*				
Negative	ref		ref	
Positive	1.73 (0.94--3.19)	0.079	1.66 (0.86--3.19)	0.129
*ypT*				
ypT0	ref			
ypT1-2	2.36 (0.68--8.15)	0.176		
ypT3-4	2.10 (0.82--5.38)	0.122		
*ypN*				
ypN0	ref		ref	
ypN1-2	2.34 (1.32--4.15)	0.004	2.11 (1.21--3.57)	0.019
*PIV*				
≤ 454.7	ref		ref	
> 454.7	3.24 (1.90--5.52)	0.002	3.08 (1.77--5.35)	0.001

CEA: Carcinoembryonic antigen; OS: Overall survival; PIV: Pan-immune-inflammation value; ref: Reference; LARC: Locally advanced rectal cancer.

**Table 4:** Univariate and multivariate Cox analysis for disease free survival in LARC

**Characteristic**	**Univariable (DFS)**		**Multivariable (DFS)**	
	**HR (95% CI)**	***P***	**HR (95% CI)**	***P***
*Age*				
< 60	ref			
≥ 60	1.18 (0.75–1.86)	0.483		
*Gender*				
Male	ref			
Female	0.94 (0.58–1.50)	0.789		
*Differentiation*				
Well	ref			
Moderate	1.19 (0.51–2.75)	0.691		
Poor	1.76 (0.62–4.94)	0.286		
*CEA pretreatment*				
Normal	ref			
Elevated	1.31 (0.83–2.05)	0.249		
*cT*				
cT1-2	ref			
cT3-4	1.01 (0.37–2.78)	0.978		
*cN*				
Negative	ref		ref	
Positive	2.03 (1.19–3.47)	0.011	2.21 (1.26–3.87)	0.006
*ypT*				
ypT0	ref		ref	
ypT1-2	1.87 (0.76–4.57)	0.171	1.86 (0.64–5.36)	0.252
ypT3-4	3.00 (1.08–8.30)	0.034	1.75 (0.82–3.74)	0.145
*ypN*				
ypN0	ref		ref	
ypN1-2	1.66 (0.99–2.78)	0.054	0.25 (0.03–1.87)	0.179
*PIV*				
≤ 454.7	ref		ref	
> 454.7	2.40 (1.52–3.78)	0.001	2.53 (1.58–4.06)	0.002

CEA: Carcinoembryonic antigen; DFS: Disease free survival; PIV: Pan-immune-inflammation value; ref: Reference; LARC: Locally advanced rectal cancer.

Table S2 displays the characteristics of the patients after propensity score matching. Univariate and multivariate logistic analyses for pCR showed that cT and PIV were important predictors for pCR, with PIV serving as an independent predictor (odds ratio ═ 0.42; 95% CI, 0.21–0.92; *P* ═ 0.041) (Table S3). Univariate Cox analyses for OS (Table S4) and DFS (Table S5) demonstrated that cT and PIV were important predictors for OS, while cT, ypN, and PIV were significant predictors for DFS. Multivariate Cox analyses indicated that PIV (HR ═ 3.11; 95% CI, 1.85–6.33; *P* ═ 0.001) and cT (HR ═ 2.51; 95% CI, 1.81–4.02; *P* ═ 0.009) were independent predictors for OS, and PIV (HR ═ 2.89; 95% CI, 1.66–5.32; *P* ═ 0.001) and cT (HR ═ 2.26; 95% CI, 1.32–4.02; *P* ═ 0.008) were independent predictors for DFS.

## Discussion

In this study, we first investigated the association between PIV and prognosis in LARC patients treated with chemoradiotherapy followed by surgery. The predictive efficiency of PIV was compared with other inflammation markers, including NLR, MLR, PLR, and SII. We observed that PIV had a better predictive ability for treatment response and survival than other common inflammation indicators and could serve as an independent prognostic factor for pCR, OS, and DFS. Therefore, PIV may be a promising inflammation marker to distinguish LARC patients with poorer preoperative chemoradiotherapy responses and long-term prognoses.

Inflammation plays a crucial role in innate immunity and is essential for immune surveillance, contributing to the elimination of external threats and safeguarding the host from potential harm [16, 17]. However, uncontrolled inflammation predisposes individuals to cancer development and promotes all stages of tumorigenesis. Within a dynamic and intricate milieu where cancer cells, stromal cells, and inflammatory cells interact, inflammation fosters processes, such as mutagenesis, cellular proliferation, and metastasis by inducing the production of cytokines, reactive oxygen species (ROS), nitrogen species, and tumor necrosis factor (TNF)-α, all of which contribute to DNA damage [18]. Elevated levels of neutrophils and monocytes within the tumor microenvironment could suppress host immunity and facilitate tumor growth by stimulating the production of myeloid-derived suppressor cells [19, 20]. Additionally, monocytes in the peripheral blood are recruited and activated within the tumor microenvironment, transforming into tumor-associated macrophages (TAMs), which play a pivotal role in promoting tumor cell invasion and metastasis through the secretion of various cytokines [21]. Platelets also play a significant role in promoting epithelial–mesenchymal transition and angiogenesis, as well as participating in thrombus formation [22]. In contrast, lymphocytes serve as essential components of cell-mediated immunity, inhibiting tumor cell proliferation and metastasis [23, 24]. Given these theories and the availability of data, compound prognostic scores calculated from peripheral blood counts, such as NLR, MLR, PLR, and SII, have been used to predict survival outcomes in cancer patients, showing good predictive ability in CRC.

Traditional inflammation indicators are typically calculated using two or three peripheral blood counts. However, the clinical utility of these inflammation-based indicators is limited due to their inconsistent ability to accurately discriminate prognoses, as evidenced by inconsistent findings in various studies [25]. Using more comprehensive inflammation indicators that reflect a broader immune-inflammatory status may enhance prognostic power. In 2020, PIV was first proposed to predict survival outcomes in patients with metastatic CRC (mCRC) [9]. This inflammation marker, calculated using neutrophil, monocyte, platelet, and lymphocyte counts, demonstrated better predictive ability for OS and progression-free survival (PFS) compared to NLR and SII in mCRC. The prognostic value of PIV has since been confirmed in non-metastatic CRC and other cancers [26, 27]. A recent meta-analysis of 1879 CRC patients showed that individuals with a low baseline PIV had better OS and PFS, despite the various cutoff values used in the included studies [28]. However, no study has previously investigated the predictive ability of PIV for tumor regression after nCRT in LARC. This study confirmed the predictive value of PIV for survival in LARC and demonstrated its good predictive ability for tumor regression following nCRT.

Several previous studies have explored the value of inflammation markers in predicting treatment responses in LARC patients undergoing nCRT. Eraslan et al. [29] found that SII, superior to NLR and PLR, had predictive power for pCR in LARC cases and could serve as an independent predictive factor. Conversely, another study observed that NLR appeared to have better predictive ability for treatment response than SII [30]. Inconsistent thresholds for indicators in various studies may contribute to this discrepancy. The present research compared the pCR prognostic value of PIV with NLR, MLR, PLR, and SII in LARC patients treated with nCRT, and PIV was found to have better predictive ability. Similarly, pre-treatment PIV demonstrated promising predictive capability for pCR and survival outcomes, surpassing the predictive performance of NLR, MLR, and PLR in Turkish women with breast cancer who underwent neoadjuvant chemotherapy [31]. In another multicenter analysis, lower levels of PIV were associated with a higher likelihood of achieving axillary pCR in patients with breast cancer who received preoperative chemotherapy [32]. Furthermore, pre-treatment PIV also proved to be an effective predictor of treatment response in esophageal squamous cell carcinoma [33], non-small cell lung cancer [34], cervical cancer [35], and stomach cancer [36] undergoing neoadjuvant treatment.

This study is the first to investigate the association between pretreatment PIV and tumor regression and survival prognosis in LARC patients who underwent nCRT. However, several limitations exist. First, selection bias and information bias are inherent in the retrospective study design. Second, although patients with hematological disorders or those receiving immunomodulatory treatments were excluded, other conditions may still influence blood-based biomarkers. Third, the limited sample size and lack of external validation may restrict the generalizability of the findings.

## Conclusion

In summary, pre-treatment PIV appears to have significant predictive value for pCR, OS, and DFS in LARC patients who received nCRT followed by surgery. Moreover, PIV demonstrates prognostic significance for survival outcomes. However, additional studies are required to validate and corroborate these findings.

## Supplemental data

Supplementary data are available at the following link: https://www.bjbms.org/ojs/index.php/bjbms/article/view/10658/3500

## Data Availability

The data that support the findings of this study are available from the corresponding author upon reasonable request.
